# From Seed to System: The Emergence of Non-Manual Markers for Wh-Questions in Nicaraguan Sign Language

**DOI:** 10.3390/languages7020137

**Published:** 2022-05-30

**Authors:** Annemarie Kocab, Ann Senghas, Jennie Pyers

**Affiliations:** 1 Departments of Psychology and Linguistics, Harvard University, Cambridge, MA 02138, USA; 2 Department of Psychology, Barnard College, Columbia University, New York, NY 10027, USA; 3 Department of Psychology, Wellesley College, Wellesley, MA 02481, USA

**Keywords:** language emergence, Nicaraguan Sign Language, non-manual markers, wh-questions

## Abstract

At a language’s inception, what determines which elements are taken up to build a grammar? How is the initial raw material reshaped through intergenerational language learning? We approached this question by focusing on the emergence of non-manual wh-question markers in Nicaraguan Sign Language (LSN), a young sign language. We asked whether the seeds of non-manual markers originate in the facial gestures of the hearing Nicaraguan community, and we explored the iterated process by which a form becomes selected and then systematized through generational transmission. We identified six non-manual facial and body movements produced with questions by 34 deaf LSN signers, representing three sequential age cohorts of learners, and compared them to those produced by 16 non-signing Spanish speakers. We examined the frequency and duration of each non-manual, and its temporal overlap with a question word. One non-manual, the brow furrow, was overwhelmingly represented among LSN signers, despite appearing rarely among non-signers and not being initially favored in duration or temporal overlap. With the second and third cohorts, the brow furrow emerges as a frequent and systematic marker. With each cycle of child learners, variable input was transformed into a more constrained set of grammatical forms.

## 1. Introduction

What are the origins of the complex symbolic systems that we find in modern human languages? Languages arise out of an interaction between the human mind that represents and organizes information, the body that manifests that information in a physical form, and the social function of transmitting the information from one individual to another. By looking at a language at its inception we can explore the nature of this interaction in order to learn about the specific contributions of these three resources. For example, what communicative expressions are pulled from the environment and how are they reshaped and reallocated as a system develops? Are initially dominant forms adopted from the outset, increasing in use as an emerging system is passed from one generation to the next? Or are alternative forms favored according to their suitability for particular functions? How is a dynamically changing linguistic system shaped according to its learnability by younger learners? In the current study we track the development of non-manual grammatical markers of wh-questions in Nicaraguan Sign Language (LSN^1^), over the course of the language’s emergence over the past 45 years. We ask whether these markers originate from co-speech facial gestures, and we test three possible determinants of their selection and systematization.

Non-manual markers in sign languages are facial and body movements, often coarticulated with manual signs, that serve linguistic functions across all levels of language, from phonology to discourse (see Sandler and Lillo-Martin 2006, for a review). Although the appearance of non-manual features in conjunction with manual signs may seem holistic to a naive eye, these non-manual elements are indeed combinatorial (Herrmann 2015; Sandler 2010). Children natively learning a sign language can readily separate non-manual features from manual features, as evidenced by their early sequential, instead of simultaneous, articulation of non-manual and manual elements (Anderson and Reilly 1997; Reilly 2006; Reilly et al. 1990).

Several researchers have speculated that the non-manuals observed in sign languages have their source in the facial gestures produced by non-signers while speaking (Benitez-Quiroz et al. 2016; Janzen and Shaffer 2002; McClave 2001; Pfau and Steinbach 2006, 2011). In at least one isolated village sign language, Kata Kolok, researchers have observed the adoption of a gestural negative headshake as a marker of negation that increases across generations of signers (Lutzenberger et al. 2022). If facial gestures are indeed a source of non-manual markers, what is the process that integrates such gestures into the grammatical system of a sign language? In examinations of other morphosyntactic structures, we have documented the emergence of simultaneous manual morphology in LSN (Senghas and Coppola 2001; Kocab et al. 2016), so we expected we might observe a similar trajectory of emergence with non-manual markers. Some evidence points to the integration of the systematic use of non-manuals over generational transmission in another recently emerging sign language, Al Sayyid Bedouin Sign Language (ABSL); only second-generation signers showed a consistent use of head and body movements that were aligned with clause boundaries (Sandler 2010; Sandler et al. 2011).

In this study, we empirically test the proposal that the facial gestures of non-signers are the source of non-manual markers in sign languages by examining the emergence of non-manual markers in LSN. We specifically focus on those non-manual markers that indicate wh-questions; that is, questions that query specific information, such as who, what, when, how many, and where. Across many different sign languages, wh-questions are accompanied by a non-manual facial gesture, most commonly either a brow furrow or a brow raise (Zeshan 2004). We extend the previous linguistic work on the emergence and grammaticalization of non-manual markers by adopting a quantitative approach to our study of the emergence of non-manuals and by explicitly looking at the rates and characteristics of the production of a variety of possible non-manuals in non-signers and in learners of an emerging sign language in Nicaragua.

The case of LSN offers a unique opportunity to test the robustness of co-speech facial gestures as a source for non-manual markers and to observe the process by which such forms are taken up and integrated into a rapidly changing linguistic system. LSN was created by deaf children and adolescents, starting with an initial cohort of 50 individuals who arrived in a new special education school in Managua in the 1970s. Although they were instructed entirely in Spanish, they communicated with each other primarily using gestures and homesigns (Kegl et al. 1999; Polich 2005; Senghas et al. 2005). Through peer interaction and intergenerational transmission, these gestures and homesigns transformed into a new, natural sign language, currently the primary, daily language of over 1500 deaf people. Because the language developed so recently, its originators are still alive today and are able to offer us a view into its origins.

Transmission from the original cohort to the learners that followed was a critical moment in the emergence of this new linguistic system. Research on artificial language emergence in the laboratory suggests that combinatoriality and systematicity can arise over repeated transmission of a system, through multiple iterations of learning (Kirby et al. 2008). In the case of LSN, the language was taken up by each cohort predominantly while they were children, who later, as adolescents, transmitted the language to a subsequent cohort of child learners. In many cases, the new arrivals introduced systematic changes to the language that the (by that time) adolescent and adult members of the community did not acquire (Senghas and Coppola 2001). We capture this change empirically, by dividing today’s LSN signers into three roughly decade-long age cohorts, with each cohort having served as a language model for the next. We can then apply an “apparent time” approach, measuring diachronic change over LSN’s first three decades with a cross-sectional comparison of these cohorts today (Bailey et al. 1991; Labov 1963; Sankoff 2006). This approach critically rests on the observation that beyond adolescence individuals do not significantly change their language, and their current language use reflects the language of their childhood (Labov 1963). In this way, we can read a layered, living record of LSN, with the first cohort’s language revealing aspects of the initial form of LSN, and the later cohorts’ representing more recent developments and changes to the system.

If the markers for wh-questions in a sign language originate in co-speech facial gestures, we should be able to find, in that seed, corresponding forms for any LSN non-manual markers we identify. Furthermore, if observable characteristics of the seed determine which forms will ultimately be taken up by the language, we should be able to capture those characteristics empirically from the outset and measure their cascading effects. We selected three candidate characteristics that might influence the adoption of a form. First, we determined how frequently the different forms are used. Previous research on older languages has shown that the frequency of a construction in learners’ input and in language-users’ production predicts language acquisition and change (Bybee 2007, 2010; Diessel 2007). Accordingly, a non-manual that appears more frequently in the facial gestures that accompany spoken questions of non-signers might be more likely to be selected as a grammatical marker of questions in LSN. Second, we considered the duration of the forms, as a measure of their fitness for acquisition and a measure of their suitability to the function of marking questions. A feature or form that is produced for a longer period has greater salience than a shorter one, making it easier to perceive and acquire (e.g., Fridland et al. 2004). Additionally, a form that can be sustained has more potential to be leveraged for its grammatical affordances such as spreading over longer phrases (Sandler 1999) as a way to mark the scope of a query. Third, we examined the timing of the non-manual, specifically whether it was produced at the same time as the question word. Wh-question non-manuals in older sign languages are consistently produced simultaneously with the manual signs for a question (Benitez-Quiroz et al. 2014). The coarticulation of the non-manual with a wh-question word might strengthen the association between the non-manual and the function of asking a question, making the mapping between form and function more salient to learners. If these characteristics drive form selection, then the facial gestures and body movements of non-signers that are most frequent, are held the longest, and overlap the most frequently with a wh-question word should be the ones taken up as non-manual markers of wh-questions in LSN.

These factors should be relevant at each moment of transmission, with each cohort seeding the next, from the hearing Spanish speakers, to the first cohort, to the second, to the third. We don’t expect the same outcome at every stage; rather, we expect the same mechanisms to apply to a dynamically changing system. As the input for each subsequent group changes, the application of these same mechanisms will yield a different, corresponding output. So, for example, we might see a tipping point where one or very few forms come to dominate. The pattern of change in the community today will give us clues to its origins. Changes that are due to adults and children using the language repeatedly, over time, should be evident in all of the groups today, since all of the members were adults at the time the recordings were made, with many years of experience using the language together. Changes that are the result of how language is learned initially by children will leave a different pattern, one of the differences between cohorts that persist in adulthood.

We might expect certain aspects of the nature of children’s learning to leave a particular kind of imprint on their language. Of course, children are not exposed directly to the grammar that produces the language they observe; instead, they observe only the patterned output that is generated by their interlocutor’s grammar. Even so, children are quite skilled at mastering the intricacies of their language. They are particularly sensitive to word-internal patterns, and detect them with very little exposure (Saffran et al. 1996). Evidence from both signed and spoken languages has shown that child learners are better than adult learners at extracting the regularities of languages (Mayberry and Fischer 1989; Saffran et al. 1996). How might we expect child learners to respond to a system that lacks such intricate regularities? One case study followed a deaf child acquiring his language from deaf parents who had learned ASL only as older adolescents, and therefore did not have the fluency of native signers. The child’s command of ASL eventually surpassed his parents’; he applied the morphology of the language, particularly the spatial morphology, more consistently than they did (Singleton and Newport 2004). In artificial language-learning experiments, when children are presented with miniature languages that are unsystematic and variable, they deviate from that input in predictable ways, increasing its regularity. Adults in the same experiments generally do not impose that kind of reorganization (Hudson Kam and Newport 2005). Given this kind of creative power in each individual learner, we were interested in discovering how repeated acquisition of LSN in its earliest years, over 1500 instances of learners, might build up the system of the language, in this case, the grammar for generating questions.

In the current study, we documented the array of facial expressions and body movements produced by Nicaraguan Spanish-speaking non-signers and computed their frequency, duration, and co-articulation with a wh-question word. We then compared non-signers’ use of these co-speech facial gestures with that of the first, second, and third cohorts of LSN signers. We hypothesized that the most frequently produced facial gestures produced by non-signers would be taken up as the preferred non-manual marker for wh-questions. We also hypothesized that facial gestures that were produced for a longer time and that were co-articulated with a wh-question word would also be more likely to be selected as a grammatical marker.

## 2. Methods

### 2.1. Participants

This study included 50 participants (23 female; 27 male) from Managua, Nicaragua. These consisted of 16 Spanish speakers who had typical hearing and no sign language experience (hearing cohort; *M*_*age*_ = 29.49, range = 17.0–55.3) and 34 deaf signers of LSN: 10 from the first cohort (*M*_*age*_ = 35.51, range = 30.5–50.0), 13 from the second cohort (*M*_*age*_ = 25.45, range = 21.5–34.1), and 11 from the third cohort (*M*_*age*_ = 20.29, range = 15.9–28.8). All LSN signers had joined the deaf community by the age of six, most upon entering school, and used LSN as their primary daily language since that time. First-cohort signers entered the community between 1974 and 1983; second-cohort signers between 1984 and 1990; third-cohort signers between 1993 and 1999. Ten additional hearing non-signers were tested but were excluded from coding due to errors in video recording. Two additional deaf signers were tested, but their data were excluded due to their history of contact with sign languages other than LSN. One first-cohort signer and four third-cohort signers were unintentionally tested at two different time points. In these cases, we selected the recording that had the best quality video for coding.

All adult participants provided verbal and written consent for participation. Participants under the age of 18 provided verbal and written assent, and their parents provided written consent for their participation. Participants were compensated for their time. Most hearing participants participated in this study only. For all deaf participants and some hearing participants, this study was conducted alongside other studies.

### 2.2. Procedure

Data collection took place between 2008 and 2017 in the city of Managua, Nicaragua and its outlying areas. Participants were seated facing a camera across from a confederate. They were instructed to ask the confederate a series of questions in order to learn specific personal information about him or her. For hearing participants, the confederate was a fluent Spanish speaker, and for deaf participants, the confederate was a fluent signer of LSN. The experimenter stood adjacent to the camera, holding up $8{\frac{1}{2}}^{\prime \prime }\;\times \;11^{\prime \prime }$ cards with brief words and phrases written in Spanish to cue the participant with the information to be elicited (Table 1). Each item was presented on a separate card. If a deaf participant did not understand the written Spanish word, the experimenter provided the LSN sign translation. Participants were never cued with the targeted question word or sign.

The initial stimulus deck used in 2008 included nine items. However, some items were not always effective at eliciting the target questions, so these items were replaced, and three items were added, for a total of twelve items for data collected in 2009 and later (Table 1). Seven first-cohort signers and eight second-cohort signers were tested using the initial 2008 stimulus deck. Sessions lasted approximately five minutes per participant and were recorded on video for later coding and analyses.

### 2.3. Coding

**Figure 1.**
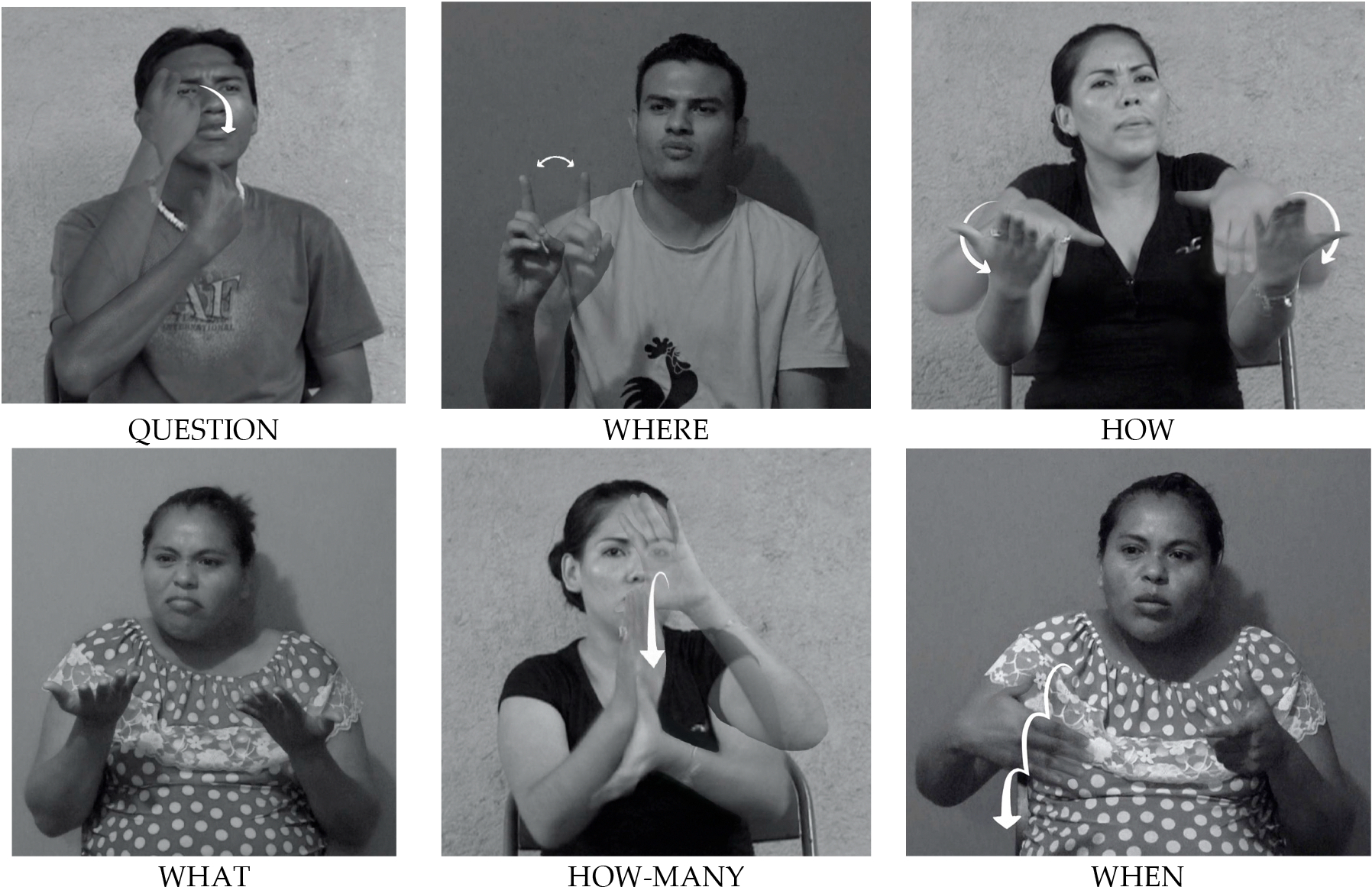
Examples of the LSN wh-question words that were elicited.

The video recordings from each session were tagged and coded using ELAN version 6.2 (ELAN 2021). Spanish data were tagged by a fluent Spanish speaker trained by the third author; LSN data were tagged by the first and third author, both fluent signers with several years’ experience coding Nicaraguan signing. Each target question was transcribed, and question words were tagged and categorized on a separate tier. Targeted question words are listed in Table 1, though all question words were tagged and coded, even if they differed from the target. Sample LSN question words are shown in Figure 1. Because we know little about the nature of the syntactic structure of questions in NSL, and even less about how this structure has changed over time, we tagged all interrogatives whether or not they included a wh-question word. Some participants asked a question more than once in response to a single prompt. Each elicited question was tagged. Once the questions were tagged, other trained coders, blind to cohort status, coded each of 6 non-manual facial expressions and body movements that had been observed with questions. Only non-manuals that overlapped with the question utterance were tagged and coded. In some cases, non-manuals began before and/or ended after the manual utterance. In rare cases, the non-manual was held through the interlocutor’s response to the question. In these cases, we computed the non-manual offset as the onset of the interlocutor’s response. Each of these non-manuals was coded on a dedicated tier in ELAN, indicating its onset and offset for every item in which it appeared. This enabled us to extract, for each non-manual, the number of times it was used, its duration, the question in which it occurred, and its temporal overlap with a targeted question word or sign. Examples of the six non-manuals can be seen in Table 2.

### 2.4. Statistics

#### 2.4.1. Dependent Variables

##### Frequency of type of non-manual use.

For each participant we computed the number of each of the coded non-manuals produced with each elicited question. This computation allows us to see the change in frequency of non-manual use across cohorts.

##### Duration of non-manual production.

For every non-manual that co-occurred with an interrogative, we measured the time from non-manual onset to offset.

##### Coarticulation of non-manuals with question words.

For the subset of questions produced with a wh-question word and with a non-manual, we categorized whether the non-manual overlapped with an overtly articulated (spoken or signed) wh-question word.

#### 2.4.2. Model Specification

For all analyses we ran mixed effect regression models using R version 4.0.3 (R Core Team 2021) and the lme4 package (Bates et al. 2015). For all models we included participants and items as random intercepts. We operationalized the change in the language by cohort. Cohort was dummy coded as hearing, first, second, and third, with hearing as the reference level. Non-manual type was dummy coded with chin lift as the reference level, because we had no theoretical motivation to preset any specific non-manual as the reference level, and that was the default reference level selected by the program. For each central question, we specify the models used.

To test the role of frequency in non-manual adoption, we ran a mixed effects linear regression predicting the total number of non-manuals produced alongside every question produced (*N* = 676) with participants and items as random intercepts, and cohort and non-manual type, and the interaction between cohort and non-manual type, as fixed factors. In addition, we ran six separate mixed effects linear regressions predicting the total number of each of the six non-manuals and cohort as a fixed factor. These analyses should allow us (a) to identify if the facial gestures of Nicaraguan Spanish speakers are the seed for the non-manual markers of wh-questions in LSN, and (b) to isolate the candidate grammatical markers of wh-questions in LSN–those that appear most frequently.

To test the role of *duration* in non-manual adoption, we ran a mixed effects linear regression predicting the duration of every non-manual produced (*N* = 1050), with participants and items as random intercepts and cohort and non-manual type, as well as the interaction between cohort and non-manual type, as fixed factors.

Finally, to capture any tendency to coordinate the timing of the non-manual with the production of a question word, we ran a mixed effects logistic regression using the glmr() function, predicting non-manual *coarticulation* with the question word, with participants and items as random intercepts and cohort and non-manual type, as well as the interaction between cohort and non-manual type, as fixed factors. For this analysis we considered only the subset of non-manuals that were produced during interrogatives where a wh-question word was also produced (*N* = 711).

When any models failed to converge, we removed participants as a random effect and changed the optimizer.

## 3. Results

Table 3 presents the total number of questions and non-manuals elicited from each cohort.

### 3.1. Frequency of Non-Manual Occurrence

To answer the question of how the available facial gestures in the hearing community have been taken up and repurposed as wh-question markers by Nicaraguan signers, we ran a mixed effects linear regression predicting total non-manual use with all questions, and then predicting the use of each of the six non-manuals (Table 4; see Statistics for model specification).

**Figure 2.**
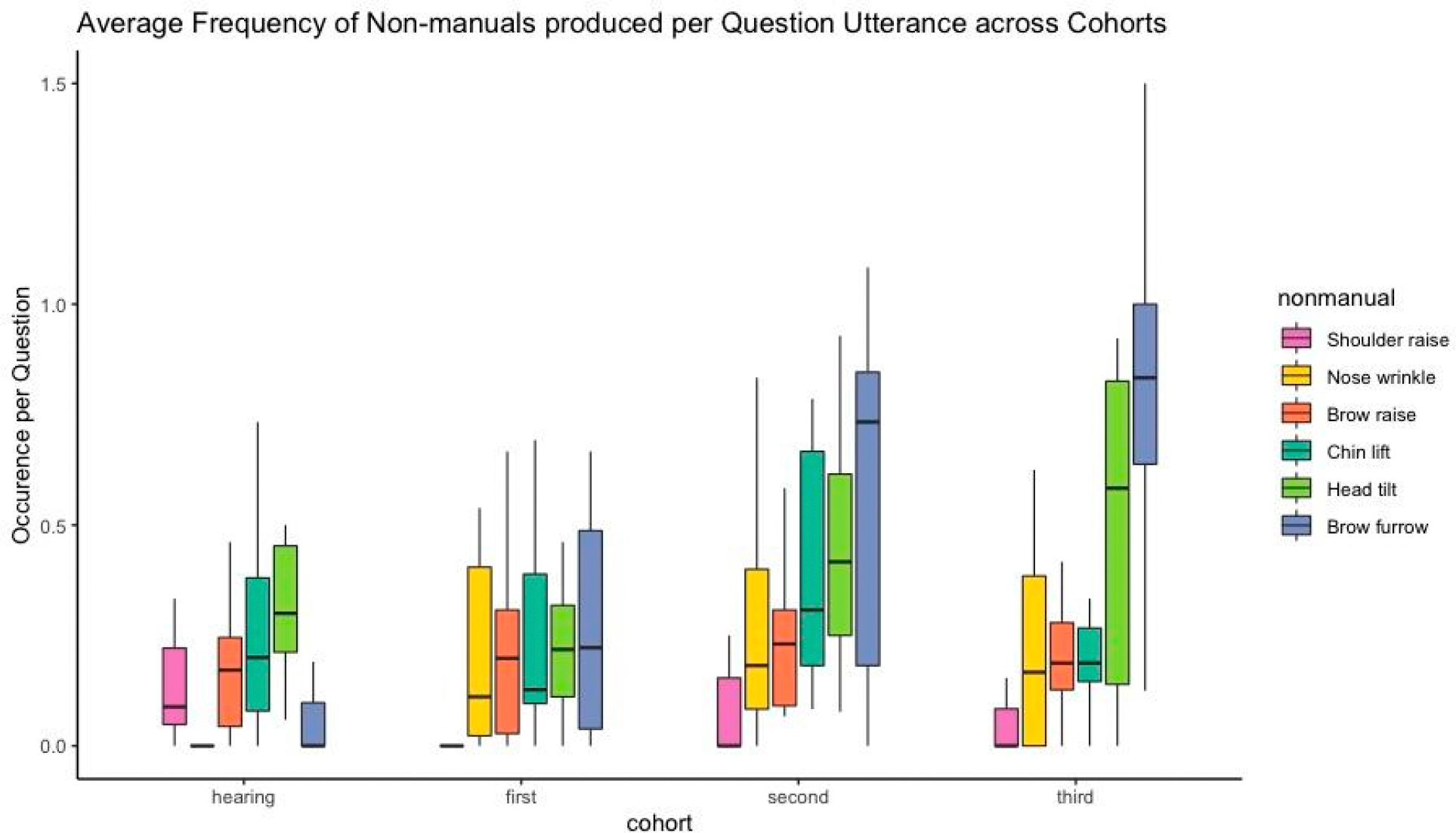
Average frequency each non-manual per question across cohorts. The black line indicates the median value for each non-manual type. Whiskers indicate the maximum and minimum values, excluding extreme outliers. Non-manuals are ordered according to mean frequency over all groups combined, with the more frequent to the right. Figure created using the ggplot2 package (Wickham 2016) in the R programming environment.

While first-cohort signers were statistically similar to the hearing non-signers in their total non-manual production, second- and third-cohort signers produced more non-manuals than the non-signer participants (Table 4). Turning to the patterns of the individual non-manuals, we see a tendency for them to increase or stabilize across the four groups, with the nose wrinkle and the brow furrow increasing to a significant degree (Table 5). Though it ultimately stabilizes at a relatively low level, the initial increase in the nose wrinkle is intriguing, given that it was virtually nonexistent among the hearing non-signers. The most dramatic change was in the use of the brow furrow, which was rare among the hearing non-signers, but came to dominate in its use by second- and third-cohort signers (Figure 2).

Visualization of the data allows us to better understand which facial gestures from the hearing community are taken up by the sign language, and how the distribution of non-manual use changes over time (Figure 2). Intriguingly, the brow furrow is the most dominant non-manual across all cohorts of signers, with a dramatic increase in its use from the first to second cohort. However, this non-manual was one of the least prevalent among the hearing participants. The non-manual most highly preferred by the hearing non-signers is the head tilt, followed by the chin lift and the brow raise. Among the signers, the head tilt emerges as the second most frequently used non-manual, but the chin lift and the brow raise remain relatively constant across groups. Thus, the primary markers of wh-questions for the later users of LSN are the brow furrow and the head tilt, even though only the head tilt, and not the brow furrow, is heavily represented among the facial gestures produced by non-signers.

### 3.2. Duration of Non-Manual

We asked whether non-manuals with the longest duration among the hearing non-signers might be especially salient, or better able to serve grammatical functions, and therefore be favored for selection. We did not observe any simple effects of group or non-manual type on duration (See Table S1 in Supplementary Materials), indicating that duration is highly unlikely to be driving the effect of cohort on frequency observed in the analyses above. What is crucial is whether we see interactive effects between cohort and non-manual type, specifically for the non-manuals that we saw most frequently among the later cohorts of signers, the brow furrow and the head tilt. Indeed, we did see an interactive effect for cohort and brow furrow, such that first and second cohort signers produced the brow furrow for a longer duration than hearing signers and relative to the chin lift (first cohort × furrowed brow: *β* = 0.63, 95% CI [0.12–1.15], *p* = 0.016; second cohort × furrowed brow: *β* = 0.65, 95%CI [0.24–1.07], *p* = 0.002). We also observed a similar interactive effect for the second-cohort signers and their production of the head tilt; they held the head tilt for longer, relative to the chin lift, than hearing signers (second cohort × head tilt: *β* = 0.39, 95% CI [0.05–0.73], *p* = 0.023).

**Figure 3.**
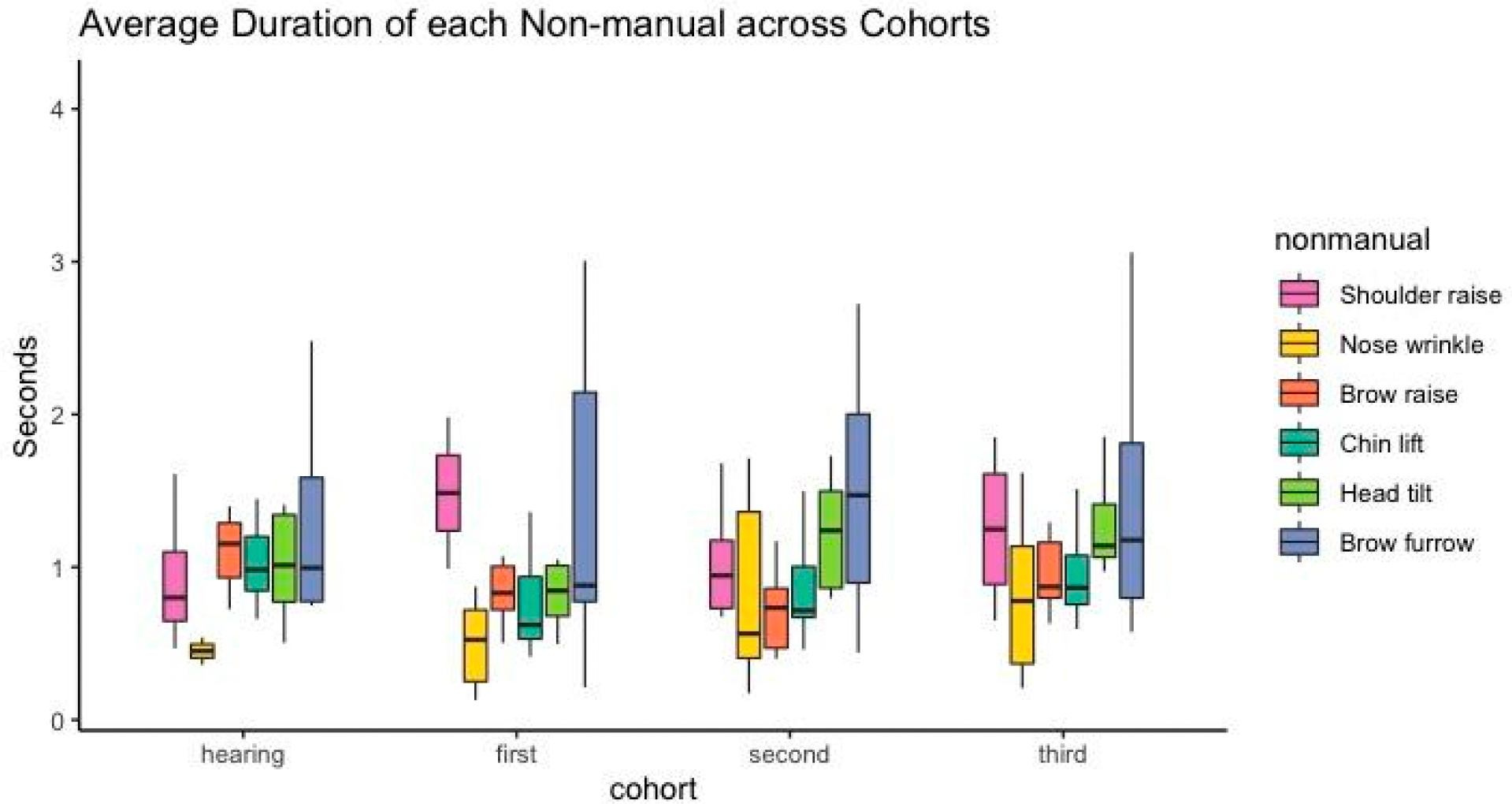
Average duration of each non-manual produced across cohorts. The black line shows median duration for each non-manual. Whiskers represent maximum and minimum scores, excluding extreme outliers. Non-manual categories are ordered according to frequency, as represented in Figure 2, with the more frequent to the right. Figure created using the ggplot2 package (Wickham 2016) in the R programming environment.

Visual inspection of the data (Figure 3) shows that among later cohorts of signers, the head tilt and brow furrow are indeed held the longest, with the brow furrow showing the greatest variability in duration. Thus, for the two non-manuals that emerge as likely candidates for grammatical markers, we observe that they are produced for a longer duration by LSN signers. The upper bounds of the whiskers in Figure 3 indicate that the brow furrow, in particular, has the potential to be held for much longer among the LSN signers, compared to the other non-manuals, and compared to the non-signers. The later emergence of a longer duration for the brow furrow suggests that duration is not driving the selection of the form; rather, it may be an indicator of increasing systematization that follows along with its increase in frequency.

### 3.3. Coarticulation of Non-Manual with Wh-Question Word

Our third test examined whether the coarticulation of a non-manual with the wh-question word made it more likely to be taken up as a marker of wh-questions. The position of the wh-word in the elicited questions varied within and across cohorts, with the wh-word appearing in sentence-initial, sentence-medial, and sentence-final positions.

We ran a mixed-effects logistic regression (see Statistics section for model specification) on the subset of the data that included the questions that had both a manually articulated question word (Figure 1) and a non-manual. The model showed that the three cohorts did not significantly differ from non-signers in their coordination of the non-manual with the wh-question word (see Table S2 in the Supplementary Materials). Regardless of cohort, head tilt was significantly more likely than the chin lift to be coarticulated with the wh-question word (*OR* = 0.39, 95%CI [0.18–0.83], *p* = 0.015). The most frequently produced non-manual, the brow furrow, was not significantly more likely than the chin-lift to be produced at the same time as the wh-question word.

**Figure 4.**
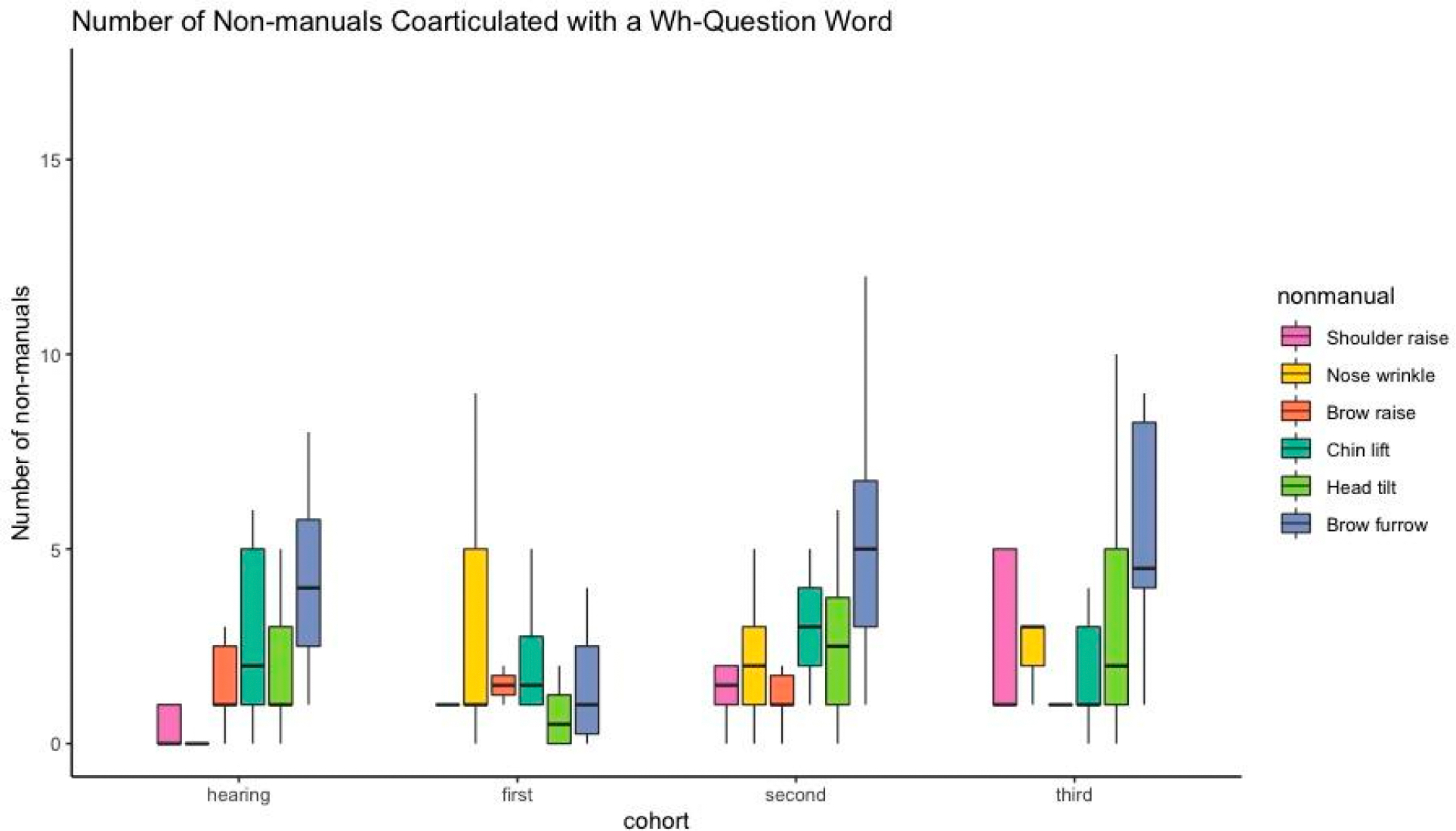
Number of each non-manuals type coarticulated with the wh-question word. The black line shows the median number of questions for which the non-manual was coarticulated with the wh-question word. Whiskers represent maximum and minimum scores, excluding extreme outliers. Non-manual categories are ordered according to mean frequency as represented in Figure 2, with the more frequent to the right. (The unusually skewed bar for the shoulder raise in the third cohort results from a single participant who repeated questions multiple times per item, raising the shoulders multiple times per question.) Figure created using the ggplot2 package (Wickham 2016) in the R programming environment.

Visual inspection of the data (Figure 4) reveals that, while on average the number of questions where the brow furrow is coarticulated with the wh-question word is relatively constant across the groups, with the later cohorts we observe more cases above the median, suggesting that within these later cohorts, more signers are articulating the question words and brow furrow simultaneously, although this effect is not significant.

## 4. Discussion

We began our inquiry into the emergence of the grammar of LSN by seeking the origins of the forms used to mark wh-questions. We looked for the seed of non-manual markers in the facial gestures of members of the hearing community as they asked questions in Spanish and followed the use of these non-manual expressions in the signing of three age cohorts of LSN, representing the first three decades of the language’s birth and growth. Our cross-sectional comparison of the frequency and duration of several non-manuals captured the progressive adoption of two of these forms: the brow furrow and the head tilt. Intriguingly, these two non-manual forms were not the ones most frequently produced by the Spanish speakers; nor were they initially sustained for the longest duration; nor were they the forms most apt to overlap in timing with the spoken wh-word. Thus, while the seed of non-manual markers can be found in the diversity of candidate facial gestures produced by the surrounding Nicaraguan hearing community, the adoption of the dominant forms into LSN is not driven by their frequency, duration, or overlap among non-signers. Rather these metrics appear to be indicators, not drivers, of language evolution.

The changes that we documented over the early years of LSN suggest a path of grammaticalization of selected non-manual forms. However, none of our three measures of the initial seed effectively determined which non-manuals would ultimately be selected for grammaticalization. We consider three possible alternative mechanisms that merit further exploration.

First, there may be other visual-perceptual or articulatory affordances, not measured in our current study, that favored these particular non-manuals for selection. For example, coarticulation with other facial gestures could work in multiple ways. The appearance of a facial gesture in isolation may make it more salient to a learner; alternatively, selection may favor facial gestures that can be easily co-articulated with other facial gestures. In addition, there are other characteristics of facial gestures, such as the intensity of the articulated form (e.g., Domaneschi et al. 2017), that may increase its salience. The affordances of particular non-manual expressions, such as the brow furrow, may allow them to be maintained over stretches of discourse. These characteristics may favor certain non-manuals in the service of prosodic functions, such as marking the boundaries of clauses and sentences, and pragmatic functions such as turn taking and back-channeling (Brentari et al. 2018; Mesch 2016; Sandler 2010; Wilbur 2013). Exploration of these other aspects of articulation would be especially informative.

Second, the selection of the brow furrow and the head tilt may have more to do with communicative salience. The brow furrow and, to a lesser extent, the head tilt, may be capitalizing on human universals in non-verbal communication. Eyebrow movements, including the brow furrow, have been observed to signal that a speaker feels uncertain or perplexed (Campbell et al. 1999; Domaneschi et al. 2017; Ekman 1979; Swerts and Krahmer 2005), two mental states that underpin requests for information. The articulatory affordance and/or communicative function of the brow furrow might explain its consistent association with wh-questions across a variety of sign languages around the world (Zeshan 2004). However, our data did not reveal a preference for hearing non-signers to produce the brow furrow with questions. Why we don’t empirically find an overwhelming preference to produce this nonmanual is unclear. The previous work showing an association between uncertainty and the brow furrow among non-signers did not measure the frequency of the production of the brow furrow with questions but rather the intensity of facial expression production (Domaneschi et al. 2017) or the interlocutor’s interpretation of these facial expressions (Campbell et al. 1999; Swerts and Krahmer 2005). The one study that quantified the number of brow furrows accompanying wh-questions produced by hearing non-signers in the U.S. also showed very low rates of production (~5% of the time; Pyers and Emmorey 2008). Thus, it may be the case that while hearing non-signers produce the brow furrow, they generally do so less often than previously estimated. Instead of the brow furrow, non-signers in the present study preferred the lateral head tilt, which also carries communicative significance. While the use of the lateral head tilt in wh-questions is less documented in sign languages, research on spoken communication has proposed that a lateral head tilt functions to make a speaker appear more friendly by offsetting direct eye contact with the listener (Costa and Bitti 2000), perhaps making a question more likely to be answered. Thus, communicative salience may account for the facial expressions that are produced by hearing non-signers when asking questions. Once a facial expression is taken up into a language, additional considerations, such as articulatory affordance, may then come into play.

Third, the gestures that hearing people produce when they speak to each other are just one of several possible seeds of LSN. It is unclear the degree to which deaf children would be able to access the ambient spoken conversations in their environment. There are likely other gestural behaviors in the communication between hearing family members and deaf children, such as enactments, pointing, and other gestures unaccompanied by speech, in the communication between hearing family members and deaf children that are taken up in the creation of the homesign systems that arise in families with a deaf child (Coppola 2020; Goldin-Meadow 2005). Indeed, parents of Nicaraguan homesigners overwhelmingly prefer to communicate with their children using manual gestures without any accompanying speech (Coppola et al. 2006). The nature of facial gestures produced without speech is an open question. However, such voice-off homesign systems were probably a more direct precursor to first-cohort signing.

Given that the brow furrow is observed in many other sign languages around the world, one might wonder about direct influence from other sign languages on LSN. Over the years, there have been several lexical signs adopted from other sign languages, some from direct contact and some via dictionaries, which we have been documenting in other studies. However, the history of language contact and the pattern of emergence of non-manuals suggest that international contact is a less likely source than local gestures. In the 1970s and 1980s in Nicaragua, and even into the 1990s, international contact was limited to a few individual signers, all members of the first cohort, for limited periods. Yet the dominance of the brow furrow is not evident in their signing. Its dominance begins with the second cohort, whose primary exposure was to that first cohort, and their own peer community. This signature suggests that it arose locally as the language was taken up by younger signers.

Whatever their pattern of initial use, the non-manuals were deployed differently by signers from different cohorts. The brow furrow appears to have shifted early from disfavor into favor. The first-cohort signers used it more frequently than non-signers, and occasionally sustained it longer (though this is not a statistically detected difference). However, the first cohort dropped any tendency to coarticulate the brow furrow with the wh-question sign. Indeed, they showed little coarticulation of any non-manual with a wh-question sign. As they took up the language, the second and then third cohorts began coarticulating the brow furrow, in particular, with wh-question signs. At the same time, they increasingly differentiated their two favored forms, the brow furrow and the head tilt, from the others in frequency. These changes over cohorts in the coarticulation of non-manual and manual signs are likely to have interacted with other changes in the use of lexical signs for questions. Consider the data in Table 3 that reveal a striking shift from first-cohort signers who rarely produced manual wh-question signs, to second- and third-cohort signers who often produced more than one manual wh-question sign in a single question. It would be a useful pursuit in future inquiry to carry out an analysis of the changing syntactic structure of wh-questions in LSN, in coordination with the changing use of non-manuals.

What does this pattern across cohorts today reveal about the nature and history of the emergence of LSN? Recall that a synchronous, cross-sectional “apparent time” analysis can be taken to reflect a diachronic history of the language, with older age cohorts reflecting language of longer ago, and younger age cohorts reflecting a more recent variant. While this pattern indicates the changes that took place, it does not fully explain why new developments in the language do not spread to all members of the community, and why an earlier variant of the language persists among older members. The three cohorts in this study roughly and categorically represent a constant, continuous influx of new members into the LSN signing community across three decades. While new arrivals learn from older members, all members continue to interact within and across age groups, and any communication between younger and older individuals would logically have an equal potential of influence in either direction. We would expect an emerging language to undergo reorganization and signal compression, in response to articulatory and perceptual pressures that favor communicative and processing efficiency (Kirby et al. 2008; Kanwal et al. 2017). However, everyone, regardless of age, is subject to these pressures. Accommodations that result during peer interaction and transmission, that are generated by or learnable by adults, should be evident in longitudinal, real-time snapshots of the language over the years, but not show up in “apparent time” cross-sectional analyses today. In contrast, differences between age cohorts that are still evident decades later point to the effect of the changing nature of learning across the life span. In such cases, their different natures will lead children and adults to arrive at different accommodations to these pressures. The measurable differences between age cohorts that we have documented, in both the selection of brow furrow by the first cohort, and the coarticulation of non-manuals by the second and third cohorts, thus reveal the nature of children’s learning in particular. Early childhood appears to be a time when key language-learning mechanisms are available, and each age cohort, in sequence, took their turn in this childhood stage, passing on a changed system.

In following these changes, we appear to have captured two tipping points in the emergence of LSN. The first, with the first cohort, entailed the selection of a small number of potential non-manual markers from the variety of facial gestures that accompanied spoken questions in the local non-signing community. As the nonmanuals were adopted into LSN, their relative frequency and use did not correspond to their use as facial gestures by the non-signers. They were less differentiated, not reflecting the variability of their source. At the same time, the non-manuals became more separate, rather than co-articulated with the wh-word in the sentence.

The second tipping point occurred when the second cohort took up LSN, and a subset of the non-manuals started to dominate. This change is particularly interesting in light of differences between adult and child learners when presented with certain kinds of variability in their input. Research using artificially created languages in the laboratory has found that when the input includes several alternative forms whose use is undifferentiated, child learners will acquire a smaller set of forms, and apply them more systematically (Hudson Kam and Newport 2005). Adult learners are not as quick to reorganize a language under the same conditions (Hudson Kam and Newport 2009). This particular solution of narrowing and systematizing appears to be an imprint of typical child learning on LSN.

As a few forms started to dominate, LSN signers of the second and third cohort increased the co-articulation of the non-manual wh-markers with the signed wh-word. The timing of this change is notable given the ecology of the language at that time. The systematization of the grammar of wh-questions in LSN happens concurrently with the establishment of a lexicon, the organization of argument structure, and the coordination of discourse, among other linguistic features. Previous work has documented that the transition from the first to the second cohort was characterized by the appearance of grammatical features that depend on simultaneous, rather than sequential, production (Senghas et al. 1997; Senghas and Coppola 2001; Senghas 2003; Kocab et al. 2015, 2016). The grammar of LSN non-manuals apparently emerged in coordination with other simultaneous aspects of the grammar.

A fuller consideration might explore these and other aspects of the changing ecology of LSN, and how the brow furrow and other non-manuals fit in as the full repertoire of signs and their syntax is being created. For example, at the transition from the first to the second cohort of LSN signers, the system for marking syntactic objects was changing (Senghas et al. 1997; Senghas 2003). Research on ASL has shown that wh-question words can be absent from the surface structure of a signed question, leaving the non-manual with a greater role in indicating the syntax of the sentence (Petronio and Lillo-Martin 1997). It would be useful to examine non-manuals’ temporal overlap with other arguments in question sentences, aside from wh-question words. We informally observed that non-manuals were sometimes co-articulated with the sign for the queried item or object. There are many such possible ways, as LSN was transmitted from one generation to the next, that the syntax of non-manual question markers may have been changing dynamically with the rest of the language.

By closely examining subtle movements of the face during a simple communication task, we have captured key aspects of the earliest stages of the evolution of markers for wh-questions in LSN. Though it represents only a sliver of the grammar, this small piece can reveal mechanisms responsible for the birth and growth of a grammar. The seeds of language are borne of countless acts of communication, in which individuals leverage the expressive power of the body to represent and transmit information. Once initial forms are available, they evolve through transmission from one person to the next. This is how a language is created. Even an element as small as the furrowing of the eyebrows, once produced, is available to be shaped into language. The changes that we have captured here reveal the progressive effects of language acquisition processes, applied by hundreds of children over several decades, with the language of the youngest LSN signers today reflecting their combined, cumulative impact.

## Supplementary Material

2022 Kocab supplementary

**Supplementary Materials:** The following are available online at https://www.mdpi.com/article/10.3390/languages7020137/s1, Table S1: Fixed and Random effects predicting non-manual duration. Table S2: Fixed and random effects predicting the coordination of the non-manual with the wh-question word.

## Figures and Tables

**Table 1.** Items of information that participants were asked to elicit from the confederate, and their respective targeted wh-question words.

Cued Information to Be Elicited	Target Wh-Question Word (English Translation)	Target Wh-Question Word (Spanish Translation)

Name	what	qué
Age	how many	cuanto
Birthday	when	cuándo
Number of siblings	how many	cuanto
Job ^1^	what	qué
Supermarket ^1^	where	donde
Favorite food ^1^	what	qué
Home address	where	donde
Friend ^1^	who, which	quién, cual
Transportation to work/school ^2^	what	qué
Mother’s birthday ^2^	when	cuándo
Waking time ^2^	when	cuándo
Dinner time ^2^	when	cuándo
Name of your teacher/boss ^2^	what	qué
Bus route nearest your home ^2^	where	donde

1 These items were included in the 2008 protocol only.

2 These items were added to the protocol after 2008.

**Table 2.** Descriptions and examples of the six non-manuals coded.

Non-Manual	Description	Example

Brow Furrow	Corresponds to Ekman’s facial action unit 4 (AU4). Comprises a pulling together of the eyebrows that often is evidenced as vertical wrinkles between the eyebrows.	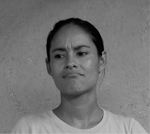
Brow Raise	Corresponds to a combination of Ekman’s facial action units 1 and 2 (AU1 + AU2) where both the inner and outer brow are raised, often resulting in lines on the forehead	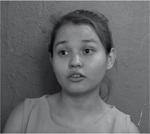
Nose Wrinkle	Corresponds with Eckman’s facial action unit 9 (AU9) which involves a pulling up of the nose and a deepening of the creases on either side of the nostrils, often with a horizontal wrinkle across the bridge of the nose	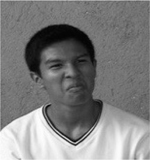
Chin lift	Operationalized as a tilting of the head backwards to raise the chin. (This movement *does not* correspond with Eckman’s facial action unit 17 “chin raiser.”)	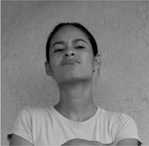
Head tilt	Any tilt of the head to the left or right of a neutral head position, also referred to as a “head cant” (e.g., Goffman 1979)	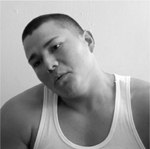
Shoulder raise	Any movement of the shoulder upwards from a neutral position; often looks like a shoulder shrug.	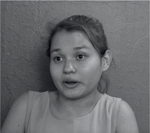

**Table 3.** Sum of questions, wh-question words, and non-manuals elicited by cohort.

Cohort	Questions	Wh-Question Words	Shoulder Raise	Nose Wrinkle	Brow Raise	Chin Lift	Head Tilt	Brow Furrow

Hearing (*N* = 16)	266	200	42	2	49	73	85	28
First (*N* = 10)	119	67	3	30	28	27	24	31
Second (*N* = 13)	165	229	12	50	39	73	73	99
Third (*N* = 11)	126	215	22	33	31	35	65	107

**Table 4.** Table of fixed and random effects predicting total non-manual production. Table generated using the tab_model function from the SJPlot package version 2.6.2 for the R programming environment (Lüdecke 2021).

	Total Non-Manuals			
	
Predictors		Estimates	CI	*p*

(Intercept)		1.03	0.64–1.41	**<0.001**
First cohort		0.28	−0.33–0.88	0.370
Second cohort		1.08	0.53–1.64	**<0.001**
Third cohort		1.22	0.64–1.81	**<0.001**
**Random Effects**				
σ^2^	1.14			
τ_00 ID_	0.49			
τ_00 item_	0.05			
ICC	0.32			
N _item_	17			
N _ID_	50			

Observations	676			
Marginal R^2^ /Conditional R^2^	0.146/0.419			

Note: lmer(total_nonmanual ~ cohort + (1 | item) + (1 | ID), data = df).

**Table 5.** Fixed and random effects predicting each nonmanual’s production. Table generated using the tab_model function from the SJPlot package version 2.6.2 for the R programming environment (Lüdecke 2021).

	Shoulder Raise			Nose Wrinkle			Brow Raise			Chin Lift			Head Tilt			Brow Furrow		

Predictors	Estimates	CI	*p*	Estimates	CI	*p*	Estimates	CI	*p*	Estimates	CI	*p*	Estimates	CI	*p*	Estimates	CI	*p*

(Intercept)	0.16	0.06–0.26	**0.001**	0	−0.11–0.12	0.941	0.19	0.10–0.28	**<0.001**	0.26	0.13–0.39	**<0.001**	0.32	0.20–0.44	**<0.001**	0.1	−0.04–0.24	0.172
First cohort	−0.14	−0.30–0.02	0.096	0.28	0.09–0.47	**0.004**	0.05	−0.10–0.20	0.494	−0.01	−0.22–0.19	0.891	−0.11	−0.31–0.09	0.271	0.18	−0.05–0.41	0.128
Second cohort	−0.09	−0.24–0.06	0.248	0.29	0.12–0.47	**0.001**	0.05	−0.08–0.18	0.459	0.18	−0.00–0.37	0.056	0.13	−0.06–0.31	0.180	0.5	0.29–0.71	**<0.001**
Third Cohort	−0.02	−0.18–0.13	0.762	0.24	0.06–0.42	**0.011**	0.07	−0.08–0.21	0.371	0.03	−0.17–0.22	0.804	0.19	−0.00–0.38	0.055	0.72	0.50–0.94	**<0.001**
**Random Effects**																		
σ^2^	0.08			0.16			0.2			0.23			0.22			0.19		
τ_00_	0.04 _ID_			0.04 _ID_			0.02 _ID_			0.05 _ID_			0.05 _ID_			0.07 _ID_		
	0.00 _item_			0.00 _item_			0.00 _item_			0.01 _item_			0.00 _item_			0.00 _item_		
ICC	0.33			0.22			0.09			0.18			0.18					
N	17 _item_			17 _item_			17 _item_			17 _item_			17 _item_			17 _item_		
	50 _ID_			50 _ID_			50 _ID_			50 _ID_			50 _ID_			50 _ID_		

Observations	676			676			676			676			676			676		
Marginal R^2^ / Conditional R^2^	0.025/0.344			0.081/0.282			0.003/0.096			0.021/0.201			0.037/0.208			0.293/NA		

Note: lmer(shoulder_raise ~ cohort + (1 | item) + (1 | ID), data = qc2); we ran this code for each non-manual type.

## Data Availability

Deidentified data presented in this study are available on request from the corresponding author.

## Footnotes

1 Following Gagne (2017) and Kocab et al. (2022), we use LSN as the initialism for Nicaraguan Sign Language, reflecting our best information about the community’s current preference for representing the name of the language: Lenguaje de Señas Nicaragüense. Previous initialisms include NSL and ISN.
