# Supplementary material for: From Seed to System: The Emergence of Non-Manual Markers for Wh-Questions in Nicaraguan Sign Language
Source: Languages (Basel). Author manuscript; Available in PMC 2025 Aug 16. (PMC12356509; doi:10.3390/languages7020137)
Supplement: 2022 Kocab supplementary [file NIHMS2100271-supplement-2022_Kocab_supplementary.pdf]

Supplementary Materials

Table S1. Fixed and Random effects predicting non-manual duration.

	Duration		
Predictors	*Estimates*	*CI*	*p*
(Intercept)	1.03	0.77 – 1.30	**<0.001**
First cohort	-0.30	-0.76 – 0.16	0.199
Second cohort	-0.14	-0.52 – 0.24	0.460
Third cohort	-0.05	-0.47 – 0.38	0.830
Brow raise	0.01	-0.26 – 0.28	0.946
Brow furrow	0.17	-0.18 – 0.52	0.330
Shoulder raise	-0.08	-0.37 – 0.21	0.580
Head tilt	0.04	-0.20 – 0.28	0.763
Nose wrinkle	-0.53	-1.54 – 0.48	0.302
First cohort * Brow raise	0.02	-0.46 – 0.51	0.922
Second cohort * Brow raise	-0.15	-0.55 – 0.24	0.440
Third cohort * Brow raise	0.15	-0.30 – 0.60	0.509
First cohort * Brow furrow	0.63	0.12 – 1.15	**0.016**
Second cohort * Brow furrow	0.65	0.24 – 1.07	**0.002**
Third cohort * Brow furrow	0.16	-0.28 – 0.61	0.473

First cohort * Shoulder raise	0.56	-0.37 – 1.48	0.237
Second cohort * Shoulder raise	0.42	-0.11 – 0.96	0.123
Third cohort * Shoulder raise	-0.02	-0.53 – 0.49	0.947
First cohort * Head tilt	0.24	-0.22 – 0.70	0.305
Second cohort * Head tilt	0.39	0.05 – 0.73	**0.023**
Third cohort * Head tilt	0.21	-0.18 – 0.59	0.296
First cohort * Nose wrinkle	0.44	-0.64 – 1.52	0.429
Second cohort * Nose wrinkle	0.49	-0.55 – 1.53	0.356
Third cohort * Nose wrinkle	0.55	-0.52 – 1.62	0.316
**Random Effects**			
σ^2	0.49		
$\tau_{00\ ID}$	0.15		
$\tau_{00\ item}$	0.00		
ICC	0.24		
N_{item}	16		
N_{ID}	50		
Observations	1050		
Marginal R^2 / Conditional R^2	0.108 / 0.318		

Note: lmer(duration ~ cohort + nonmanual + cohort*nonmanual + (1|item) + (1|ID), data=nm)

Table S2. Fixed and random effects predicting the coordination of the non-manual with the wh-question word.

	wh_coordination		
Predictors	*Odds Ratios*	*CI*	*p*
(Intercept)	2.26	1.22 – 4.20	**0.010**
First cohort	1.32	0.31 – 5.61	0.704
Second cohort	1.00	0.43 – 2.37	0.991
Third cohort	0.83	0.30 – 2.31	0.726
Brow raise	0.97	0.36 – 2.59	0.953
Brow furrow	2.57	0.65 – 10.08	0.176
Shoulder raise	0.46	0.18 – 1.17	0.102
Head tilt	0.39	0.18 – 0.83	**0.015**
Nose wrinkle	0.00	0.00 – Inf	0.991
First cohort * Brow raise	0.21	0.02 – 1.97	0.173
Second cohort * Brow raise	0.42	0.09 – 1.87	0.252
Third cohort * Brow raise	0.25	0.05 – 1.32	0.102
First cohort * Brow furrow	0.18	0.02 – 1.57	0.120
Second cohort * Brow furrow	0.92	0.18 – 4.79	0.922
Third cohort * Brow furrow	0.84	0.15 – 4.55	0.838
First cohort * Shoulder raise	4258877.93	0.00 – Inf	0.988
Second cohort * Shoulder raise	3.85	0.56 – 26.41	0.170

Third cohort * Shoulder raise	26.49	2.48 – 282.70	**0.007**
First cohort * Head tilt	0.48	0.06 – 3.95	0.495
Second cohort * Head tilt	2.94	0.92 – 9.42	0.069
Third cohort * Head tilt	2.86	0.79 – 10.39	0.110
First cohort * Nose wrinkle	7549915.23	0.00 – Inf	0.991
Second cohort * Nose wrinkle	8665736.83	0.00 – Inf	0.991
Third cohort * Nose wrinkle	19270962.97	0.00 – Inf	0.991
**Random Effects**			
σ^2	3.29		
$\tau_{00\ item}$	0.13		
ICC	0.04		
N_{item}	16		
Observations	711		
Marginal R^2 / Conditional R^2	0.308 / 0.334		

Note: glmer(wh_coordination ~ cohort*nonmanual + (1|item), data=nm_wh, family=binomial, nAGQ=0, glmerControl(optimizer = "bobyqa", optCtrl = list(maxfun=2e5)))

.
